# Endoscopic submucosal dissection of a quasi-circumferential lesion of the ileo-cecal valve by using a novel adjustable traction device

**DOI:** 10.1055/a-2051-8765

**Published:** 2023-03-30

**Authors:** Jean Grimaldi, Louis-Jean Masgnaux, Timothée Wallenhorst, Romain Legros, Jérémie Jacques, Jérôme Rivory, Mathieu Pioche

**Affiliations:** 1 Gastroenterology and Endoscopy Unit, Edouard Herriot Hospital, Hospices Civils de Lyon, Lyon, France; 2 Gastroenterology and Endoscopy Unit, Pontchaillou University Hospital, Rennes, France; 3 Gastroenterology and Endoscopy Unit, Dupuytren University Hospital, Limoges, France

The technique of submucosal dissection has been expanding rapidly for several years thanks to numerous technical advances. One of these major advances is the improvement of traction strategies, and in particular the appearance of the multi-traction technique. However, several locations remain challenging because of their anatomical particularities, especially lesions of the ileo-cecal valve, which have been the subject of the development of different strategies in recent years 1 2 .

We have developed an intensity-modulated multitraction device 3 4 5 , the A-TRACT 2 + 2 ( Fig. 1 ), which seems very promising for ileo-cecal valve lesions because it allows good exposure of the ileal part of the lesion.

**Fig. 1:**
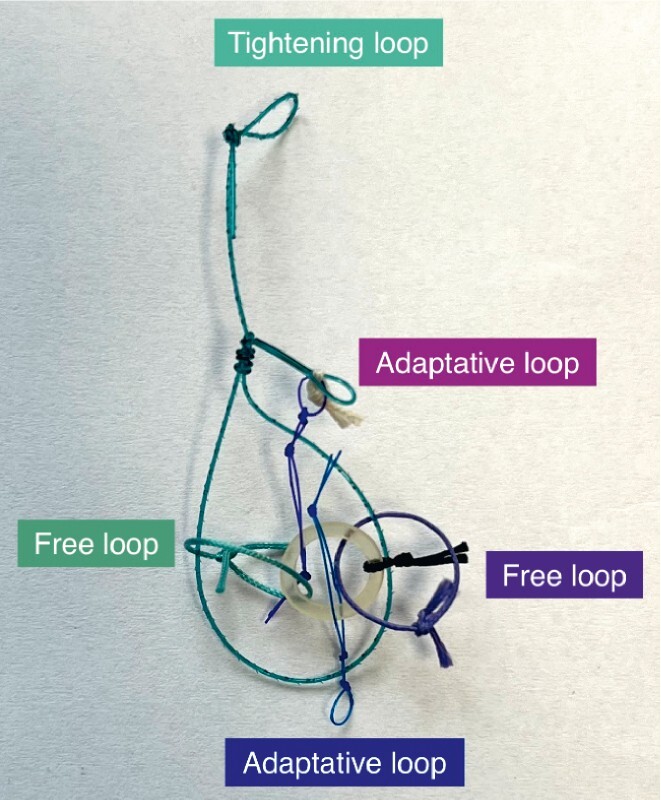
The A-TRACT 2 + 2 device.

We report here the case of a 79-year-old patient referred for resection by submucosal dissection of a quasi-circumferential lesion of the ileo-cecal valve, measuring 6.5 × 4.5 cm ( Video 1 ). After making the circumferential incision, we placed the two adjustable loops of the device on the cecal side of the lesion, allowing the start of the dissection on this side ( Fig. 2 ). In a second step, we placed the two free loops on the ileal side of the lesion and fixed the elastic band on the cecal wall opposite the lesion, allowing good traction force to continue the dissection on the ileal side.

**Video 1** Endoscopic submucosal dissection of a quasi-circumferential lesion of the ileo-cecal valve by using a novel adjustable traction device.


**Fig. 2:**
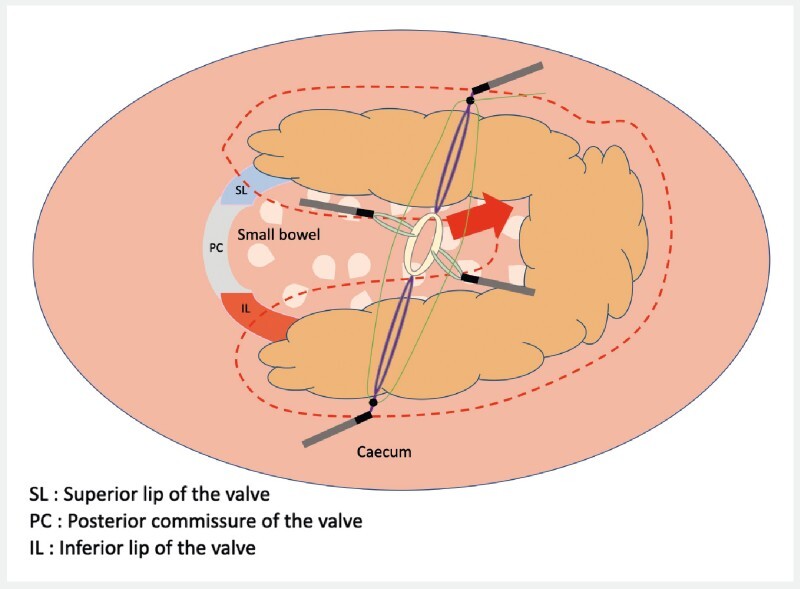
Schematic representation of the lesion and the valve seen from the front after placement of the four loops on the edges of the lesion. The two adaptable loops are attached to the cecal part and the two free loops are attached to the ileal part.

Finally, after 2/3 of the dissection, the traction force having clearly decreased due to the flexibility of the valve tissues, we re-tensioned the device to obtain excellent exposure and allow completion of the dissection ( Fig. 3 ).

**Fig. 3:**
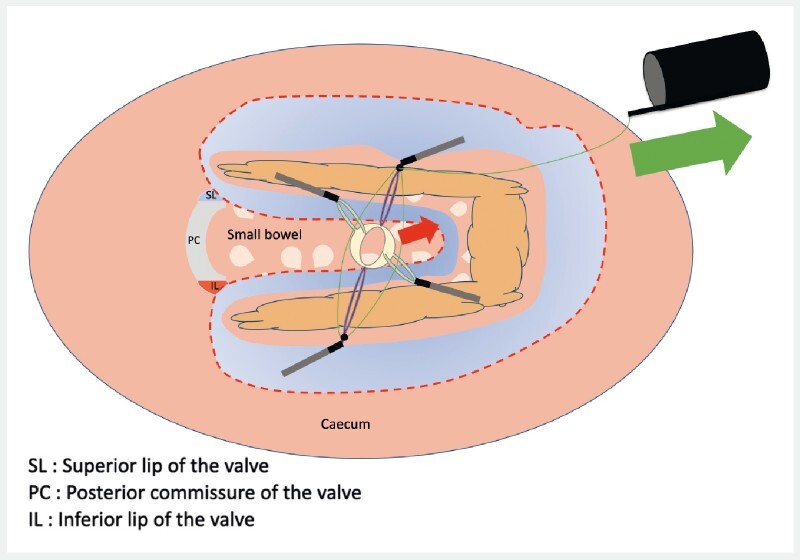
Schematic representation of the lesion and the valve seen from the front after tightening the device, allowing excellent exposure of the sub-mucosae.

To limit the risk of stenosis as much as possible, a strip of healthy ileal mucosa was preserved during the procedure. In addition, we placed the scar closure clips to keep the valve open and obtain a directed wound healing to limit the risk of stenosis.

This technique allowed an R0 resection of the lesion. There were no complications during the procedure.

Endoscopy_UCTN_Code_TTT_1AQ_2AD

## Notes

Endoscopy E-Videos

https://eref.thieme.de/e-videos
*Endoscopy E-Videos* is an open access online section, reporting on interesting cases and new techniques in gastroenterological endoscopy. All papers include a high quality video and all contributions are freely accessible online. Processing charges apply, discounts and wavers acc. to HINARI are available. This section has its own submission website at https://mc.manuscriptcentral.com/e-videos
